# Our Book Table

**Published:** 1885-11

**Authors:** 


### OUR BOOK TABLE.

Quiz Questions. Course on Dental Pathology and Therapeutics, Philadelphia Dental College: By Prof. J. Foster Flagg, D. D. S. Answered by William C. Foulks, D. D. S. Third edition, revised and enlarged. Philadelphia: The S. S. White Dental Manufacturing Company, 1885.

The world has a liking for the sententious. Pithy, terse, laconic sayings, apothegms, aphorisms, axioms, will be treasured in the memory when the learned and ponderous treatises are forgotten. Proverbs do not always give the whole truth, but what they do tell is in such a compact form that it is ever before the mind's eye. The pointed, expressive questions in the book under notice, with the brief but significant answers, convey a world of information. Like a dictionary, there is an immensity of reading matter, though it is not always connected. It would be impossible to cover, in the ordinary exegetical manner of writing, a tithe of the subjects concerning which this book gives information.

We are not an adherent of the pathological theories of Prof. Flagg, nor are we always clear concerning his scientific accuracy; we think he sometimes confounds cause with effect, and takes narrow views of many broad questions. He is, in our opinion, too apt to seize a single phase of a subject, and from it draw definite conclusions which are at variance with the matter as a whole. But no one ever denied that he was an earnest and vigorous thinker, or that he had not the courage of his convictions, and did not express himself in a clear, concise, and energetic manner. He is emphatically one of those writers whom we delight to read—and to differ with. The general tone of the book is as positive, as incisive, as is Prof. Flagg himself, and a careful study of it will benefit any thinking man. Of its popularity, the three editions found necessary to supply the demand are quite sufficient evidence.

The publishers have issued it in a very handsome manner, blank leaves for annotations being left opposite each page. The typography is delightfully clear and readable.

VERNON GALBRAY, or the Empiric. The History of a Quack Dentist. Second edition. London: E. T. Whitefield.

When this book was first published it received a great deal of attention at the hands of the dental profession. Nor has the interest in it yet died out. The copies have been carefully hoarded until it is almost impossible to find one of the original edition. It was published anonymously, but to-day it is no secret that it was written by that Nestor of professional journalism, Felix Weiss, L. D. S., of London. The narration is singularly clear and well sustained, and it details all the artifices and chicanery to which charlatans resort to obtain practice. It is entertaining reading for a dentist, for some of the devices to which the hero of the tale had recourse are very ingenious indeed.

NOTES FROM A DENTIST'S CASE-BOOK. By FELIX WEISS, L. D. S., R. C. S. Reprinted from *The British Journal of Dental Science.* London: J. & A. Churchill.

This little work, first printed some years ago, is what its name indicates—notes upon cases in actual practice, by a close observer and a concise and lucid writer—one who has the faculty of seizing upon the salient and material points of a practical instance, and drawing from them the moral which they should teach. Many of the cases detailed may be studied with great profit.

LETTERS OF A MOTHER TO A MOTHER ON CHILDREN'S TEETH. By "Mrs. M. W. J." Third edition, revised and enlarged. Philadelphia: The Welch Dental Co., 1885.

We have, before this, expressed in an unmistakable way, our approval of these letters. We can say nothing in addition to our words of commendation of the former edition of this valuable little work. The readers of the INDEPENDENT PRACTITIONER, in almost every number for some time past, have had ample evidence of the talent which the author possesses for catching the striking points in a dental essay or discussion, and that same faculty has enabled her to present in this book those vital facts which it is so essential for all mothers to know. This edition is a great improvement upon some of those previously issued, and does its enterprising publishers credit.

SCIENTIFIC ADAPTATION OF ARTIFICIAL DENTURES. By DR. C. H. LAND, Detroit, Mich.

This gives the author's views of the philosophy of the adhesion of suction plates, with directions for making a successfully fitting denture. It also contains some very useful hints for laboratory practice.

THE MEDICAL PRESS OF WESTERN NEW YORK.

We have received the first number of a monthly journal with this name, to be published in Buffalo by an association of medical practitioners. It will be edited by Roswell Park, M. D., Professor of Surgery in the Medical Department of the University of Buffalo, assisted by Prof. M. D. Mann, of Buffalo, Ely Van De Warker, M. D., of Syracuse, and W. J. Harriman, M. D., of Rochester.

The first number gives promise of unusual excellence. Not only is it a very handsome journal, but the articles which it contains are of a very high order of merit. Prof. Park is unusually well qualified for his position. He is not only a terse and vigorous writer, of great learning and enthusiasm, but he has also had valuable editorial experience, having formerly been the editor of that sterling journal, *The Weekly Medical Review. The Medical Press* starts off with the brightest prospects, as the association which publishes it is composed of about one hundred physicians, all of whom have made cash subscriptions to a permanent fund for its sustenance. There is no doubt that it will at once assume a high place in medical literature. Intending subscribers should address A. M. Barker, M. D., 137 West Tupper St., Buffalo, N. Y.

We have also received the following books and pamphlets:

*Transactions of the Michigan Dental Association. Twenty-ninth and Thirtieth Annual Sessions.*

*Quarterly Bulletin of the New York Post-Graduate Medical School and Hospital.* Aug., 1885.

*The Therapeutics of High Temperatures in Young Children.* By William Perry Watson, A. M., M. D. Reprinted from *The Archives of Pediatrics* of September, 1884.

*An Address on Cholera Infantum.* By William Perry Watson, A. M., M. D. Reprinted from *The Archives of Pediatrics* of August, 1885.

*Tracts on Massage.* Translated from the German of Reibmayr, with notes, by Benjamin Lee, A. M., M. D., Ph. D.

*Shadows in the Ethics of the International Medical Congress.* By Levi Cooper Lane, A. M., M. D.

*Brow Presentation.* With Reports of Cases. By Eli H. Long, M. D. Lecturer on Special Therapeutics, University of Buffalo. Reprinted from the *American Journal of Obstetrics* of September, 1885.

*Address of the State Board of Health of the Commonwealth of Pennsylvania.*

*Voice in Singers.* Read before the Ohio State Medical Society, by Carl H. Von Klein, A. M., M. D.

*Clinical Notes on the Local Treatment of Disease.* A record of Practical Therapeutics. By Charles L. Mitchell, M. D.

*Tabular Statistics of One Hundred Cases of Urethral Stricture treated by Electrolysis, without relapse.* By Robert Newman, M. D. Reprinted from *The New England Medical Monthly.*

*Duty of the State toward the Medical Profession.* By Conrad George, M. D. Reprinted from *The Physician and Surgeon* of July, 1885.

*Notes on the Use of Cocaine in Hay Fever.* By Robert Bartholow, M. D., LL. D.

*Report of the Proceedings of the Tennessee State Board of Health,* 1885.

*Second Annual Report of the Iowa State Board of Dental Examiners.*

*Das Fullen der Zahne mit Gold, etc., nach deutscher methode. Von Wilhelm Herbst, Zahnarzt, in Bremen.*

A number of New Medical Journals have been received, which will receive notice in due time.

